# Pros and cons of an aggressive initial treatment with surgery and radioiodine treatment in minimally invasive follicular thyroid carcinoma

**DOI:** 10.1186/s13044-022-00143-3

**Published:** 2023-01-16

**Authors:** Elisa Minaldi, Carlotta Giani, Laura Agate, Eleonora Molinaro, Rossella Elisei

**Affiliations:** Department of Clinical and Experimental Medicine, Unit of Endocrinology, University of Pisa, via Paradisa 2, 56124 Pisa, Italy

**Keywords:** Follicular thyroid carcinoma, Radioiodine ablation, Thyroglobulin, Biochemical recurrence

## Abstract

**Background:**

Currently, surgery alone is the gold standard treatment for minimally invasive follicular thyroid cancer (mi-FTC).

**Case presentation:**

A case of a mi-FTC diagnosed in 1994 was treated with total thyroidectomy and radioiodine (RAI) ablation, according to the therapeutic algorithm used at that time. Nevertheless, he had a recurrence with distant metastasis after 24 years from the initial treatment.

**Conclusion:**

Total thyroidectomy and RAI ablation might have delayed the development of distant metastasis but they were not sufficient to avoid disease recurrence. Certainly, remnant ablation simplified the follow-up and the monitoring of serum thyroglobulin allowed the early detection of the biochemical recurrence, but didn’t change the outcome of the disease. Moreover, because of this early detection the patient was exposed to useless biochemical and imaging examinations. The aim of this report is to discuss the pros and cons of an aggressive treatment of a patient with mi-FTC.

## Background

Differentiated thyroid cancer (DTC) has generally a good prognosis, with a 5-year survival reaching the 98.4% [1]. Nevertheless, persistence or recurrence can be observed with a different prevalence varying from 3% in low-risk up to 68% in high-risk group, according to the level of risk stratification [2]. In the past, when there was a high prevalence of DTC patients with advanced disease at initial diagnosis, total thyroidectomy and cervical lymph node dissection were recommended in patients with palpable lymph node metastases and radioiodine (RAI) treatment in patients with tumors > 1.5 cm [3, 4]. This “complete” approach (total thyroidectomy and RAI ablation) was adopted in subsequent years in order to eradicate normal thyroid remnants and to achieve undetectable Thyroglobulin (Tg) levels, thereby facilitating the detection of recurrent/persistent disease during follow-up. While improvements in disease-free survival and disease-specific mortality following RAI ablation have been clearly demonstrated in high-risk patients [5–9], in low-risk patients RAI ablation has shown no benefits in terms of mortality rates and inconsistent results in terms of recurrence rates [10–15]. In line with these studies, the current international guidelines recommend a less aggressive surgical treatment for low-risk DTC, such as lobectomy or active surveillance [16, 17], and discourage RAI ablation [16]. Nevertheless, this approach has been, and still is, widely debated. In 2019, a joint statement called “the Martinique Principles” acknowledged the absence of high-quality evidence on this matter and concluded that the decision to perform RAI ablation should take into account the individual risk factors, such as postoperative risk assessment, as well as patient-related factors (comorbidities, emotional concerns) and the healthcare environment (e.g., availability and quality of ultrasound evaluation, Tg measurement, RAI imaging, surgeon expertise) [18].

We hereby report a case of a minimally invasive follicular thyroid cancer (mi-FTC) diagnosed in 1994, that today we would have treated just surgically, perhaps with lobectomy. The patient was instead treated with total thyroidectomy and high-activities of RAI for remnant ablation, according to the therapeutic algorithm used at that time. The aim of this report is to evaluate the pros and cons of an aggressive treatment of a patient with mi-FTC.

## Case presentation

In January 1994, O.M., a 50-year-old male patient underwent a left lobectomy for a thyroid nodule. Histologic examination revealed a follicular adenocarcinoma, 6 cm in size, Hürthle cell variant, with minimal invasion of the tumor capsule. As consequence of the unexpected histological result, in March 1994 he underwent a completion thyroidectomy and histologic examination was negative for cancer cells. In April 1994, he underwent a high-activity radioiodine treatment (150 mCi) to remove post-surgical thyroid tissue, both normal and tumoral if present. Post therapeutic whole-body scan (WBS) showed only a neck uptake referred to residual thyroid tissue. In 1995 and 1996, two diagnostic WBS were performed in hypothyroidism after discontinuation of levo-thyroxine therapy and showed no uptake. Tg serum levels were 0.0 mcg/L in 1995 and 0.76 mcg/L in 1996. Until 2014, the patient was clinically examined annually, with neck ultrasound and laboratory testing of Tg, which revealed levels under 1 mcg/L. From 2014 to 2018 he did not seek any more medical examinations.

**Fig. 1:**
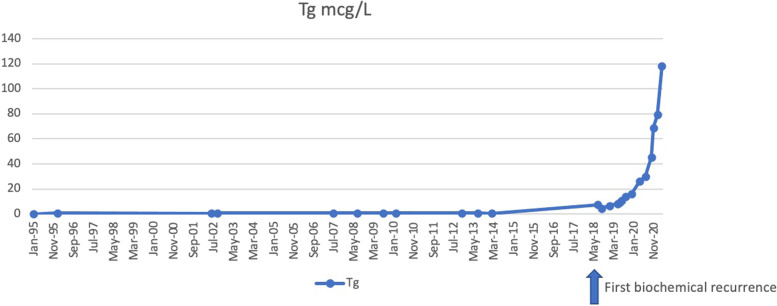
Thyroglobulin (Tg) level changes over time. Tg was undetectable until 2018, when it started to exponentially increase

From May 2018, Tg levels began to gradually increase (4.4 mcg/L) (Fig. 1). Therefore, a second treatment with high activities of radioiodine (130 mCi) after levothyroxine withdrawal was administered in October 2018. Tg levels in hypothyroidism (TSH 60 mUI/L) were still elevated (Tg 17 mcg/L). Post-therapeutic WBS showed no uptake of radioiodine. In July 2019, Tg was 11 mcg/L with TSH 0.02 mUI/L, thus he underwent a PET 18-FDG scan which revealed a 6 mm pulmonary nodule in the right superior lobe with SUV = 7.47. However, the CT scan performed shortly thereafter was negative. After 6 months, Tg was 16 mcg/L with TSH 0.02 mUI/L. PET-18FDG was repeated and confirmed the presence of the pulmonary nodule in the right superior lobe (7 mm ex 6 mm) and detected 2 more millimetric lesions.

**Fig. 2:**
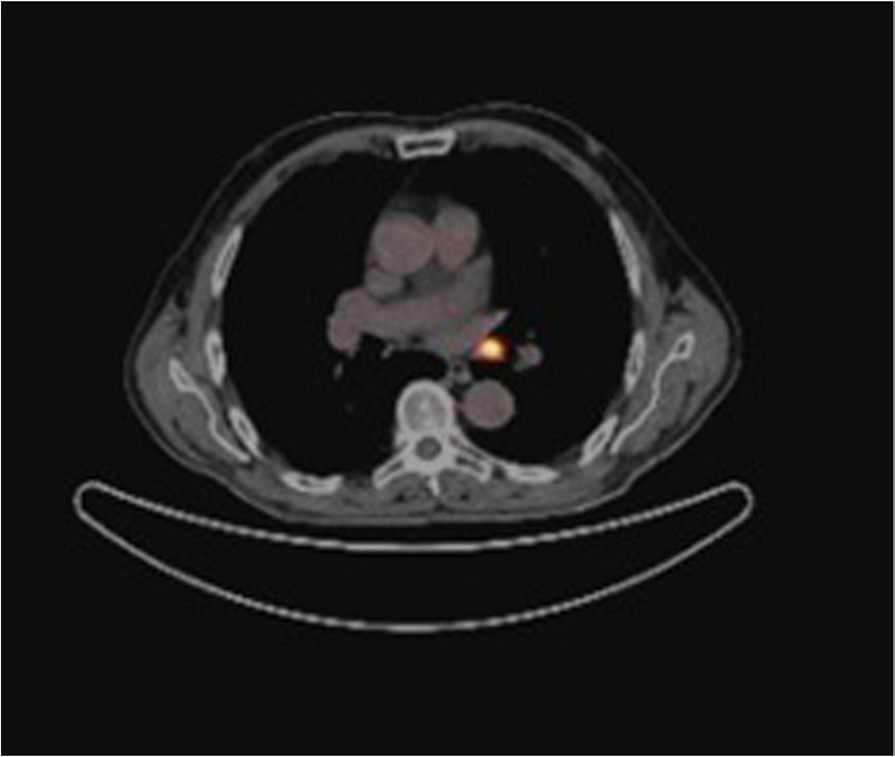
18FDG- PET/TC scan performed in February 2021 showed the increase of both dimension (12 mm ex 6 mm) and FDG-uptake (SUVmax = 27.2 ex 8.6) of the known lesion at the left inferior lobar bronchus

In March 2020, the patient underwent another radioiodine treatment (150 mCi) after levothyroxine withdrawal. Tg levels in hypothyroidism (TSH 73.3 mUI/L) were still elevated (Tg 54.4 mcg/L). Post-therapeutic WBS showed no radioiodine uptake. A second CT scan was again negative for distant metastases; thus, the pulmonary nodule has been followed-up with PET-18FDG exams. The last exam, performed in February 2021, showed a progressive increase of the lung metastasis (the larger was 12 mm ex 6 mm) and of the intensity of the FDG uptake (SUV = 27.2) (Fig. 2).

So far, from May 2018 Tg levels are continuing to increase up to 118.3 mcg/L (Fig. 1). At this point he arrived at our first observation: he is now under active surveillance and, whenever a progression of the disease will be demonstrated according to RECIST [19], we will start with a systemic therapy.

## Discussion and conclusion

According to the 2015 American Thyroid Association (ATA) guidelines, RAI treatment is recommended for high-risk DTC, it should be considered for intermediate-risk DTC and it should not be routinely administered in low-risk patients [16]. The European Association of Nuclear Medicine disagreed with this recommendation, claiming that in low-risk patients there is a lack of solid evidence for the actual benefit of surveillance over RAI ablation [18, 20]. In particular, the issue is whether a patient who does not receive a “complete” treatment (total thyroidectomy and RAI ablation) is exposed to the risk of a late diagnosis of persistent/recurrent disease [21]. Although this is not our personal experience [22], some authors argued that 14.4% of low-risk patients with undetectable Tg and Tg antibodies had still radioiodine-avid metastatic lesions detected on post-therapeutic imaging 3–4 months after surgery and that this percentage should not be neglected [23].

We reported the case of a mi-FTC treated with the “complete” approach, according to the guidelines in force in 1994, relapsed after 24 years from the initial treatment, that today we would have likely treated just with surgery.

FTC is considered at low risk of recurrence if “minimally invasive”, namely an intrathyroidal tumor with capsular invasion and no or minimal (< 4 foci) vascular invasion. On the contrary, an FTC with extensive vascular invasion should be considered at high risk of recurrence and treated with RAI [16]. Based on the current risk stratification, our patient with a mi-FTC should not had undergone RAI treatment. Nevertheless, by using the “complete” therapeutic approach (total thyroidectomy and RAI ablation), which was in line with the recommendations of those years, the patient somehow had a benefit. Thanks to the periodic assessment of Tg levels, an early detection of the disease recurrence was possible. We might speculate that if he had been treated only with total thyroidectomy or simple lobectomy, a slight raise of Tg levels, that in our case flagged the disease recurrence, would have remained unacknowledged for some time, maybe some years. In this hypothetic scenario, we probably would have blamed the physician who made the decision on the initial treatment without RAI ablation. However, considering the early detection of the recurrence, a real benefit for this patient is debatable. From the first raise of Tg levels in 2018, the patient intensified the frequency of his medical checks and underwent two more high-dose RAI treatments, of which the second was definitively unnecessary and the first was ineffective because of the radioiodine-refractoriness. He also performed many imaging exams in search of the disease location. Over a 3-year period, he had a total of 3 CT scans and 4 PET-18FDG exams that eventually detected 3 millimetric lesions in the lung. Moreover, he was submitted to two useless 131-I treatments since it was already demonstrated that the disease was radiorefractory.

In the hypothetic scenario of an initial treatment with surgery only, the Tg increase would anyway happen and thus considered unequivocally suspicious for disease recurrence. At that point a CT scan would be performed and lung metastasis would be revealed, likely at the time to be treated.

Going back to the real case report, the “complete” treatment with surgery and RAI ablation did not spare the patient from the long-term recurrence. There are no studies available on recurrence rates specifically for FTC, treated with or without RAI. The evidences available focus on DTCs in general and, anyway, rarely consider homogenous and properly stratified populations [24]. Available data specifically on FTC concern mortality rates and found no significant differences in survival if RAI ablation is performed [25, 26]. In particular, a SEER registry secondary analysis did not find any improvement of the disease-specific survival in patients with FTC < 1 cm treated with RAI ablation in a multivariate analysis adjusted for age, histology, disease extent, type of surgery, and external beam radiation therapy [25]. However, this study focused just on small FTC (< 1 cm) and included some cases with histological aggressive features and thus not considerable as low-risk tumors. Another recent retrospective study made on 858 FTC and 476 Hürthle cell thyroid carcinoma, both < 1 cm, did not demonstrated any survival benefit in patients treated with RAI [26].

Two prospective trials, ION (NCT01398085) and ESTIMABL2 (NCT01837745) are ongoing with the purpose to compare the outcomes in low-risk DTC, including FTC, patients treated with RAI ablation. Data from ESTIMABL2 trial drawn after 3 years from the randomization showed no differences in number of tumor-related events that led to perform a subsequent RAI treatment between patients in the follow-up group (4.4%) and in the ablation group (4.1%) [15]. A long follow-up of at least 10 years will be needed in order to provide a strong and reliable evidence [20]. Otherwise, we would need to wait the long-term follow-up of low-risk FTC patients that today are not treated with RAI ablation in accordance to the 2015 ATA guidelines.

Another issue to be discussed is the dimension of the primary tumor. In this case report, the recurrence, albeit late, occurred in a patient with a 6-cm intrathyroidal tumor. It might be hypothesized that a such high dimension could enhance the risk of recurrence. Some authors suggested that, similarly to papillary thyroid cancer, in FTC there is a difference in outcome according the tumor size, although using different cut-offs [27, 28]. Sugino et al. found that in FTC the risk factors for distant metastasis during follow-up were age and a primary tumor size > 4 cm [27]. Similarly, Goffredo et al. found that a larger tumor size was correlated with a more frequent vascular invasion [30]. Nevertheless, 2015 ATA guidelines still consider FTC > 4 cm at low risk as long as it does not have a widely invasion of tumor capsule and extrathyroidal extension [16].

In conclusion, we cannot exclude that in our patient the RAI ablation might have delayed the development of the distant metastasis but certainly it was not sufficient to avoid the disease recurrence. Moreover, if it is true that the remnant ablation simplified the follow-up and allowed the early discovery of the biochemical recurrence, it did not change the outcome of the disease.

Furthermore, it has not yet been proven that RAI ablation has an effective impact on patient’s outcome and that the delayed detection and treatment of persistent/recurrent disease can reduce the chances of recovery in DTC patients who have not been RAI-ablated. From a practical point of view this case shows that the outcome of this patient was not really due to the type of treatment chosen but to the biological behavior of the tumor, likely already determined at the time of diagnosis.

## Data Availability

Not applicable.

## Abbreviations

ATA: American Thyroid Association
DTC: Differentiated thyroid cancer
FTC: Follicular thyroid carcinoma
mi-FTC: Minimally invasive follicular thyroid carcinoma
RAI: Radioiodine
Tg: Thyroglobulin
WBS: Whole-body scan

## References

[1] SEER Cancer Stat Facts: Thyroid Cancer. National Cancer Institute. Bethesda MD. Available from: https://Seer.Cancer.Gov/Statfacts/Html/Thyro.Html. [Accessed: 1 Sept 2022].

[2] Tuttle RM, Tala H, Shah J, Leboeuf R, Ghossein R, Gonen M (2010). Estimating risk of recurrence in differentiated thyroid cancer after total thyroidectomy and radioactive iodine remnant ablation: using response to therapy variables to modify the initial risk estimates predicted by the new american thyroid association st. Thyroid.

[3] Mazzaferri EL, Young RL, Oertel JE (1977). Papillary thyroid carcinoma: the impact of therapy in 576 patients. Med (United States).

[4] Mazzaferri EL, Jhiang SM (1994). Long-term impact of initial surgical and medical therapy on papillary and follicular thyroid cancer. Am J Med.

[5] Taylor T, Specker B, Robbins J (1998). Outcome after treatment of high-risk papillary and non-Hurthle-cell follicular thyroid carcinoma. Ann Intern Med.

[6] Shoup M, Stojadinovic A, Nissan A (2003). Prognostic indicators of outcomes in patients with distant metastases from differentiated thyroid carcinoma. J Am Coll Surg.

[7] Durante C, Haddy N, Baudin E (2006). Long-term outcome of 444 patients with distant metastases from papillary and follicular thyroid carcinoma: benefits and limits of radioiodine therapy. J Clin Endocrinol Metab.

[8] DeGroot LJ, Kaplan EL, Straus FH (1994). Does the method of management of papillary thyroid carcinoma make a difference in outcome?. World J Surg.

[9] Tsang RW, Brierley JD, Simpson WJ, Panzarella T, Gospodarowicz MK, Sutcliffe SB. The effects of surgery, radioiodine, and external radiation therapy on the clinical outcome of patients with differentiated thyroid carcinoma. Cancer. 1998;82(2):375–88. 9445196

[10] Sawka AM, Thephamongkhol K, Brouwers M (2004). A systematic review and metaanalysis of the effectiveness of Radioactive Iodine Remnant ablation for well-differentiated thyroid Cancer. J Clin Endocrinol Metab..

[11] Jonklaas J, Sarlis NJ, Litofsky D, Ain KB, Bigos ST, Brierley JD (2006). Outcomes of patients with differentiated thyroid carcinoma following initial therapy. Thyroid.

[12] Sacks W, Fung CH, Chang JT (2010). The effectiveness of radioactive iodine for treatment of low-risk thyroid cancer: a systematic analysis of the peer-reviewed literature from 1966 to April 2008. Thyroid..

[13] Schvartz C, Bonnetain F, Dabakuyo S (2012). Impact on overall survival of radioactive iodine in low-risk differentiated thyroid cancer patients. J Clin Endocrinol Metab.

[14] Dehbi HM, Mallick U, Wadsley J (2019). Recurrence after low-dose radioiodine ablation and recombinant human thyroid-stimulating hormone for differentiated thyroid cancer (HiLo): long-term results of an open-label, non-inferiority randomised controlled trial. Lancet Diabetes Endocrinol..

[15] Leboulleux S, Bournaud C, Chougnet CN (2022). Thyroidectomy without Radioiodine in patients with low-risk thyroid Cancer. N Engl J Med.

[16] Haugen BR, Alexander EK, Bible KC (2016). 2015 american thyroid Association Management Guidelines for adult patients with thyroid nodules and differentiated thyroid Cancer: the american thyroid Association Guidelines Task Force on thyroid nodules and differentiated thyroid Cancer. Thyroid.

[17] Molinaro E, Campopiano MC, Elisei R (2021). MANAGEMENT OF ENDOCRINE DISEASE: papillary thyroid microcarcinoma: toward an active surveillance strategy. Eur J Endocrinol.

[18] Michael Tuttle R, Ahuja S, Avram AM, Bernet VJ, Bourguet P, Daniels GH (2019). Controversies, Consensus, and collaboration in the Use of 131I Therapy in differentiated thyroid Cancer: a Joint Statement from the american thyroid Association, the European Association of Nuclear Medicine, the Society of Nuclear Medicine and Molecular I. Thyroid.

[19] Eisenhauer EA, Therasse P, Bogaerts J, Schwartz LH, Sargent D, Ford R (2009). New response evaluation criteria in solid tumours: revised RECIST guideline (version 1.1). Eur J Cancer.

[20] Verburg FA, Aktolun C, Chiti A (2016). Why the European Association of Nuclear Medicine has declined to endorse the 2015 american thyroid Association Management Guidelines for adult patients with thyroid nodules and differentiated thyroid Cancer. Eur J Nucl Med Mol Imaging.

[21] Hindié E, Taïeb D, Avram AM (2018). Radioactive iodine ablation in low-risk thyroid Cancer. Lancet Diabetes Endocrinol.

[22] Agate L, Bianchi F, Brozzi F, Santini P, Molinaro E, Bottici V (2019). Less than 2% of the low- and intermediate-risk differentiated thyroid cancers show distant metastases at Post-Ablation Whole-Body scan. Eur Thyroid J.

[23] Campennì A, Giovanella L, Pignata SA (2018). Undetectable or low (< 1 ng/ml) postsurgical thyroglobulin values do not rule out metastases in early stage differentiated thyroid cancer patients. Oncotarget..

[24] Lamartina L, Durante C, Filetti S (2015). Low-risk differentiated thyroid cancer and radioiodine remnant ablation: a systematic review of the literature. J Clin Endocrinol Metab..

[25] Kuo EJ, Roman SA, Sosa JA (2013). Patients with follicular and hurthle cell microcarcinomas have compromised survival: a population level study of 22,738 patients. Surgery.

[26] Khokar AM, Holoubek SA, Kuchta KM (2020). Survival with follicular and Hurthle Cell Microcarcinoma compared to papillary thyroid microcarcinoma: a Population Study of 84,532 patients. World J Surg.

[27] Asari R, Koperek O, Scheuba C (2009). Follicular thyroid carcinoma in an iodine-replete endemic goiter region: a prospectively collected, retrospectively analyzed clinical trial. Ann Surg.

[28] Ito Y, Hirokawa M, Higashiyama T (2007). Prognosis and prognostic factors of follicular carcinoma in Japan: importance of postoperative pathological examination. World J Surg.

[27] Sugino K, Ito K, Nagahama M (2011). Prognosis and prognostic factors for distant metastases and tumor mortality in follicular thyroid carcinoma. Thyroid.

[30] Goffredo P, Jillard C, Thomas S (2016). Minimally invasive follicular carcinoma: predictors of vascular invasion and impact on patterns of care. Endocrine.

